# 

**Published:** 1904-02-20

**Authors:** 


### CONTENTS.

**Medicine and the War** - 365

**Hospitals and General Practitioners** - 366

**Annotations** - 367

**Notes and News** - 368

**Hospital Clinics and Medical Progress—**

The Degenerate Infant - 369

On Boldness in the Treatment of Heart Disease - 370

Ovarian and Pelvic Dermoids - 370

The Early Diagnosis of Consumption - 371

The After-history of Gastric Ulcer - 371

Traumatic Hæmatorrhachis - 372

PROGRESS IN BACTERIOLOGY - 372

PROGRESS IN SURGERY OF THE LIVER, GALL BLADDER, AND SPLEEN 373

PROGRESS IN FEVERS - 374

**The Book World of Medicine and Science** 376

**Hospital Administration—**

Historical Sketch of Some of the London Hospitals.—II. - 377

British Health Stations.—II. - 379

The Story of the Insane from Year to Year - 379

Practical Departments - 381

**Editor's Letter-Box** - 382

**The Student of Medicine** - 382

*NURSING SECTION.*

**Notes on News from the Nursing World—**

Queen Alexandra's Military Nursing Service—The Queen's Commemoration Fund—The Russian Sick and Wounded—No Foreign Nurses for Japan—Lord Dudley and St. Patrick's Nurses—Parliament and State Registration—The Standard of Mental Nursing—District Training at Keighley Hospital—One Nurse for 98 Cases—Reported Dismissals at Tooting Bec Asylum—"In the Garb of a Professional Nurse"—The Memorial Home at Tunbridge Wells—A Nurse and her Notice—A new Movement in Wiltshire—The Holt-Uckley Nurses—Another District Nurse wanted at Swansea—Promptitude in District Nursing—Nurses' Weddings - 279–282

**The Nursing Outlook** - 283

**Lectures on Ophthalmic Nursing** - 284

**The Nurses of Leavesden Asylum for Imbeciles** 285

**The Preparation and Application of an Abdominal Fomentation** - 287

**Locked in a Mortuary** - 288

**Appointments** - 289

**Death in Our Ranks** - 289

**Everybody's Opinion** - 290

**Presentations** - 290

**Echoes from the Outside World** - 291

**Notes and Queries** - 292

**For Reading to the Sick** - 292

